# The Relationship between Emotional Intelligence and Psychological Well-Being among Male University Students: The Mediating Role of Perceived Social Support and Perceived Stress

**DOI:** 10.3390/ijerph17051605

**Published:** 2020-03-02

**Authors:** Romualdas Malinauskas, Vilija Malinauskiene

**Affiliations:** Department of Physical and Social Education, Lithuanian Sports University, Sporto 6, 44221 Kaunas, Lithuania; vilija.malinauskiene@lsu.lt

**Keywords:** emotional intelligence, cross-lagged, perceived well-being, perceived social support, perceived stress

## Abstract

This study aimed to examine the subject of emotional intelligence (EI), which has received increased attention from scholars over the past few decades. The study utilized a quantitative longitudinal approach to attain the objective of understanding the correlation between EI and psychological well-being. A sample consisting of only male students was sought in this study in a process that was guided by specific criteria. The study reveals that students’ EI correlates positively with perceived social support and well-being at each time and across times. Negative relations are found between perceived stress and well-being at each time and across times. Results and findings reported in this study reveal that perceived social support partially mediates the longitudinal association between EI and well-being. Specifically, perceived stress does not mediate the longitudinal association between EI and well-being.

## 1. Introduction

In the past few decades, there has been an increased interest in the subject of emotional intelligence (EI). Research studies have adopted various approaches to understanding the relationship between EI and other variables, such as psychological well-being, which has also garnered interest from scholars. Evidence reported in such studies has described the correlation between EI and well-being [1], as well as with other variables [2,3]. However, findings from existing studies have reported unclear evidence on the issue surrounding EI and its relative connection to the psychological mechanism [3]. The current study, which has adopted a longitudinal approach, has sought to examine the existing relationship between EI and psychological well-being. Additionally, the relationship was pursued by exploring perceived social support and perceived stress as the mediators of the relationship. The longitudinal approach spans two time points to provide clear relevant methodological suggestions that ascertain the patterns evident in the relationships between the four variables identified in this case.

### 1.1. A Theoretical Model for Examination

**Figure 1:**
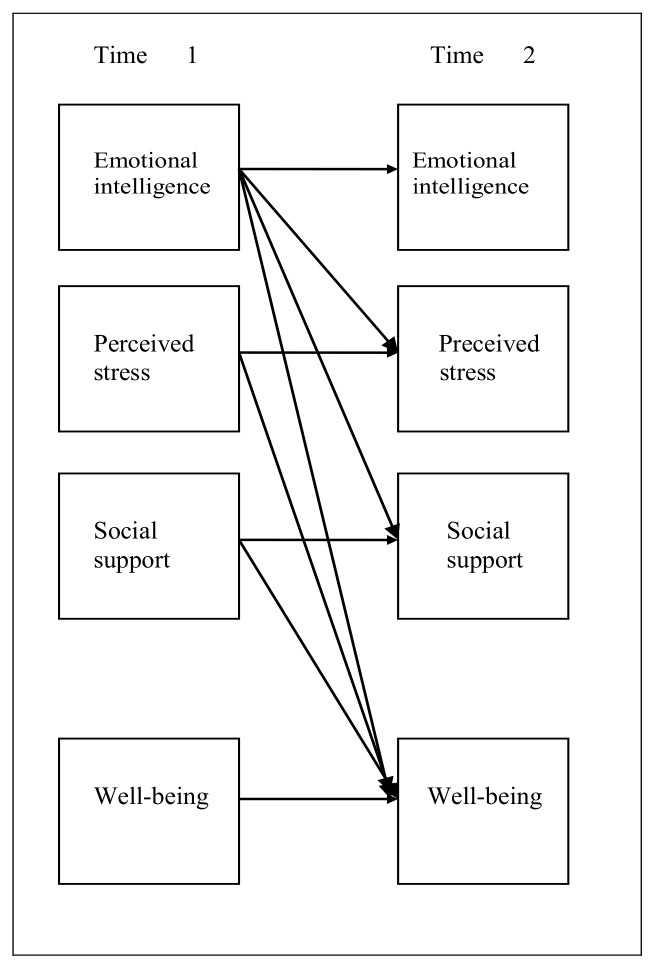
A theoretical model for examination.

The figure below demonstrates the conceptual model embraced for this investigation. As outlined in Figure 1, the conceptual model highlights the two timeframes between which the theoretical and empirical evidence from previous studies are structured. The figure outlines the theoretical model that provides an understanding of the relationship between EI and the variables sought in this study [4,5,6,7].

Use of the longitudinal approach, showcased in the figure above, is key to understanding the relationship between the variables within the structured timeframes. In terms of understanding emotional intelligence and psychological well-being, it is vital to understand the various definitions of the two concepts that are used. This is because the concepts are used frequently when determining the existing relationships between the variables and EI. Whereas the term “psychological well-being” remains unknown [8], findings from a study conducted by Diener and Oishi [9] recognize that positive experiences linked with the absence of negative experiences define the well-being of an individual. Conversely, findings reported by Ryff and Keyes [10] note that psychological well-being can not only be defined within the parameters of positive and negative effect but also as the whole state of being free from stress and not experiencing any psychological problems. Additionally, evidence from studies conducted on psychological well-being indicates that the concept consists of varying empirical and theoretical indicators [11]. Therefore, the measures identified above are key to determining the overall score of psychological well-being. The adoption and use of Ryff’s psychological well-being concept in this study is based on its major trait as a confounding multidimensional model.

As a concept that continues to attract research, EI is acknowledged by scholars for providing the necessary skills that are relevant for addressing the well-being of individuals [12]. Evidence reported in the scientific literature conceptualizes EI using either trait or ability approaches, ascertained through trait EI and ability EI methods of assessment, respectively. Whereas trait EI focuses on emotion-related dispositions that are associated with self-perceptions measured using self-reporting [13], ability EI deals with emotion-related cognitive abilities that are measured using maximum performance tests [14].

The present study focuses on the trait EI technique due to its straightforward way of being evidenced with measurements that are actualized using numerous instruments [15]. Additionally, the rationale for selecting this approach is that trait EI outlines a set of behavioral dispositions and self-perceptions that are concerned with an individual’s ability to comprehend and utilize emotional information. Another significance is that trait EI can be applied with ease in a social context [16]. The selection of this approach was also guided by the expected outcome of good discriminant validity and values that demonstrate predictive validity, exemplified in the case of EI [17]. The application of the trait EI instrument derived from the Schutte Self-Report Inventory (SSRI) [1], which draws ideas from the original model developed by Salovey and Mayer [18], in the present study was because it was tested and validated using a Lithuanian-speaking sample [4].

Findings from conducted studies have expounded on the relationship of people’s well-being with the concerned variable, EI [19,20,21]. Persons who identify their emotional issues can enjoy better psychological well-being regardless of the value of their EI [12,22]. Existing evidence demonstrates an increased validity of EI in controlling personality and determining well-being [23,24], perceived stress [25,26,27], and social support [23,28,29]. Despite many studies that point to the correlation of EI to well-being, only a few longitudinal studies have taken place [30,31]. Cross-sectional studies do not provide much evidence about causality [7]. As a result, the approach used in this study will guide us in establishing the relationship between EI and the key variables sought in this study.

Despite existing scientific literature being clear in associating EI with psychological well-being and well-being, there is a gap that fails to highlight the process of the relationship [32]. There is insufficient evidence pointing to the potential mediating variables accounting for the relationship between psychological well-being and EI. A case in point, a study by Zeidner, Matthews, and Roberts [22], shows mediating variables influencing the well-being–EI relationship. However, social support serves as a potential variable linking EI and well-being/psychological well-being [2]. Different scholars state that social support is vital for well-being [9].

Researchers have found that emotional intelligence results in the acquisition of social skills, which results in enhancing the availability of social support, a pre-requisite of well-being [33]. Based on the above, several possible explanations better describe the situation. First, EI is developed because of excellent social skills; existing evidence shows that high scores in EI are associated with social competence. [34]. Furthermore, EI is linked to greater availability of social support, not to mention higher satisfaction and contentment in receiving social support [35]. Ideally, EI can result in an individual getting more social support because, as a variable, it is a reflection of acquired social skills, such as skills relevant in obtaining social support, developed through experiencing meaningful social interaction [36]. Interestingly, individuals’ social skills resulted in getting social support from members within their social networks. It translates to better social support; as a variable, social skill is an important mediating variable that justifies how higher well-being is influenced by social support [37].

People with high EI tend to receive more significant social support [2,23,29]. Furthermore, research in different capacities has demonstrated that people who receive sufficient support from others in social settings experience a better sense of well-being and satisfaction [7,23]. Scholars have found that perceived social support serves as a partial mediator, which sets the stage for the relationship between well-being and emotional intelligence [23] and life satisfaction [7]. Conclusively, it is ideal for expecting that perceived social support serves as a mediator linking EI and psychological well-being.

Perhaps another potential variable worth investigating in the mediating relationship between EI and well-being is perceived stress. The main objective of this study focuses entirely on perceived stress. For instance, perceived stress weighs heavily on an individual’s feelings in the face of perceptions of stressful experiences. Therein, the implication is that accrued stress levels can have an impact on individual experiences and emotional well-being. Several scholars report that perceived stress can impact an individual’s well-being adversely in instances where these individuals perceive the situation as stressful and are unable to handle a disturbing environmental stimulus [38]. Correspondingly, life satisfaction and personal happiness are a result of the influences of perceived stress, particularly at an individual level [39].

A closer examination of variables demonstrates that perceived stress mediates the relationship between EI and well-being indicators regarding life satisfaction and happiness [3]. Past studies have suggested that EI minimizes perceived stress and all the consequences associated with negative stress [29]. Scholars have shown that EI is associated with regulating the emotions and lowering the levels of perceived stress [40,41] and that EI allows individuals to manage their stress levels better [42,43]. Researchers have suggested that EI serves as a mechanism to predict perceived stress based on how individuals responded to stimuli [27].

Several scholars have theorized in cross-sectional studies that EI has an impact on the level of perceived stress when predicting well-being among students [5,6]. Researchers hold conflicting viewpoints on the existence of a middle ground concerning cross-sectional analysis [44]. This implies that in a longitudinal study, a mediator has significant weight in the effects and causes of the relationship between different variables. Therefore, we maintain the perspective that mediation as a process takes place gradually over time [45]. The process on its own requires a sequential alignment of independent variables that comes before the mediators, preceding the desired outcomes. In line with this logic, one can hypothesize that perceived stress results in the mediation of a time-specific correlation between EI and well-being. The same occurrence is applicable in perceiving social support through the interaction of emotional intelligence when contrasted to well-being.

Nonetheless, EI was pivotal in the wellness of the male students at the educational institution. Findings from other studies have revealed that female students’ EI averages better than their male counterparts [46]. Notably, researchers stipulate that difference in sexes of the students has an impact on their perception of stress and well-being in school [47]. The unique choice of men in this study can be justified by the fact that previous studies have supported the idea that males experiencing great social support were more satisfied with their lives compared to females [2,48]. The evidence suggest [2] that male students experience higher stress levels and are considered to be unhappy most of the time due to increased stress levels. Some evidence [49] further shows that EI is responsible for males experiencing adverse psychological outcomes. It is useful to study the relationship between EI and psychological well-being in male students because at high levels of EI indicators, male university students have reported lower health behavior than female university students at the same level of EI indicators [4]. It was also decided that only men should participate in the study because, in developing stress management programs for increasing well-being, empirical research has shown that training in emotional regulation (training for developing EI) may be more beneficial for males than females [19].

Finally, there is a need to learn and research further to better understand the male’s viewpoint in responding to stressful circumstances or situations [48]. Different students state that they have experienced varying types of stress, hence the discrepancies in their need for social support, depending on the individual. Therefore, having a better understanding and knowledge of EI will be ideal in helping students transform their weaknesses into strengths and overcoming challenges they face, hence improving their well-being [50].

Some scholars have proven [2,49,51] that university students state that they need social support because of increased stress levels. In retrospect, this shows the correlation between an individual’s emotional wellness and emotional intelligence. However, in a longitudinal study, bias arises because of the parameters that are conditioned to meet the objectives of the researcher [52], as opposed to addressing the perceived stress among the male population in the context of university students. The current study doubles up as a narrative because it is based on the ideas arising when investigating the correlation between EI and well-being in the male athletic student population. However, it fails to account for the distinct characteristics associated with a male student population in a university.

### 1.2. Significance of Study and Hypotheses

The aim of this study is to examine the existing relationships between EI and both confounding and mediating variables, such as perceived social support and stress. Even though meta-analyses have reported persuasive evidence for the EI and well-being relationship through cross-sectional studies [31,53], there is as yet no clear evidence from longitudinal studies of the direct and indirect effects of EI on well-being through perceived stress or perceived social support. Similarly, evidence from most studies on EI’s and psychological well-being’s relationship based on a cross-sectional study design has yielded concerns about the causality effects. This study examines the causal relationships formed from the EI–well-being relationship. For instance, evidence derived from the male population has failed to substantiate specific characteristics experienced in a population consisting of male university students.

Hypothesis 1: Using the longitudinal approach, perceived stress at Time 1 is associated with EI at Time 1 and well-being at Time 2. This hypothesis is drawn from an earlier study that evidenced the mediation of perceived stress between EI and well-being indicators, such as satisfaction and happiness [3]. While EI was categorized under skills, findings reported that EI was responsible for lessening the perceived stress.

Hypothesis 2: Perceived social support at Time 1 mediates in a longitudinal association between EI at Time 1 and well-being at Time 2. This hypothesis is also based on previous research studies that revealed how social support mediated the relationship between EI and psychological well-being [2] and that social support was central for well-being [9].

## 2. Materials and Methods

### 2.1. Study Design

The design of this study involved a quantitative longitudinal approach. The study was planned to take at least three months, with the lag expected to be covered in two specific timelines. One reason for choosing this lag time was the relevance of the longitudinal mediation model in measuring the relationships between the variables, including EI, perceived social support, and perceived stress.

### 2.2. Participants

The sample size for this study was established with support from the Paniotto formula for the male population [54]. The population of the male students was computed by *n* = 1/(Δ2 + 1/N). In this case, n signifies the sample size, while Δ represents the margin of error, which in this case is 5%. When calculating the estimates, we need to take into consideration the sample size of the town, which we estimate to be 20,000, represented by N.

Therefore, the computation was as follows: *n* = 1/(0.0025 + 1/20,000) = 392. This study used cluster sampling to recruit study participants. Study participants were recruited across higher learning institutions from a list of 15 universities. The three universities were selected by simple random sampling. Students from each of the three universities were selected by selecting every male student from each university roster (*N* = 604). Study participants participated in the survey using a paper-and-pencil test. The sample size was calculated to ensure that the gap left by those who failed to complete the survey was addressed. Study participants who failed to complete the survey were excluded from the study. The remaining 437 male university students were involved in the study, which was defined by the two timeframes.

### 2.3. Instruments

Emotional intelligence is measured using the SSRI, which was validated by Schutte et al. [55]. The SSRI is commonly referred to as the emotional intelligence scale, the Schutte Emotional Intelligence Scale (SEIS), or the SEI (Self-Report Emotional Intelligence) and measures EI by testing the students rigorously. Technically, the methodology utilized here measures how participants perceive stressful situations and how it affects their emotions. It makes use of quadrants in understanding the emotional perceptions of the participants; these quadrants are good emotional experiences, adjustment of emotional experiences in a social setting, use of emotional experiences, and the ability to express feelings to oneself and others. The quadrants are composed of 33 items, measured on a five-point scale (1 = strongly disagree, 2 = disagree, 3 = neutral, 4 = agree, and 5 = strongly agree). We opted to analyze data without the use of subscales because they would interfere with the outcomes obtained.

The sum of the emotional intelligence is calculated by obtaining the average of the outcomes in the items specified by the participants. Internal consistency was estimated to be good and expressed as α = 0.86. Notably, the Lithuanian version of the SSRI shows 0.79 as the constant value, which is confirmed through further tests [4] and gives a test–retest reliability coefficient of 0.84 for the overall questionnaire [4]. Comparisons of the overall questionnaire scores confirm the absence of significant mean difference and small effect size (Cohen’s d = 0.09) between the English and Lithuanian versions of the SSRI.

The Multidimensional Scale of Perceived Social Support (MSPSS) serves as the other tool that is relevant in the study. The MSPSS proved ideal for gauging participants’ perceptions of social support [56]. The MSPSS is psychometrically sound and is useful in constructing adequate construct validity and factor validity and excellent reliability [56]. This tool is used because it ensures that researchers work within economies of scale when taking measurements. The tool in question has 12 descriptive items that have three different subscales. It deals with support from the participants’ families, friend support, and significant other support. The MSPSS also adheres to the use of specific terms regarding the descriptions of important supporting individuals. It is designed to allow the respondents to interpret items in the most relevant manner. For example, things measured through the support of a significant other are referred to as “special person”. This term could mean a boyfriend, girlfriend, counselor, teacher, among many others. The respondents used a seven-point Likert-type scale (very strongly disagree to very strongly agree) in which they expressed their interest for each item.

Scholars point out that the accuracy and correctness of the scale used shows a level of satisfaction [56]. Additionally, the Cronbach’s alpha was noted to be 0.71 and extended to 0.69, 0.74, and 0.76. Subsequently, the subscales used show support from important persons that are able to give the necessary support to the individuals during tests. The Lithuanian version of the MSPSS has an internal consistency of 0.61. Equally, the present study indicates that perceived social support can be obtained by averaging the items.

The Perceived Stress Scale-10 (PSS-10): The tool in question encompasses 10 items [57]. It has a self-report inventory that assesses the degree to which situations within an individual’s life change due to stressful circumstances. Overall, the study design was focused on the PSS-10, which evaluates the responses of the participants in terms of their unpredictability, control, and overload of work that affects their personal emotions in response to events and situations. The PSS-10 is ideal for acknowledging that these three issues form the central component of personal experience in terms of stress-related problems. Based on these three issues, the respondents expressed how they thought about a problem or what they felt, and their emotion was measured on a five-point Likert scale (0 = never, 1 = rarely, 2 = sometimes, 3 = fairly often, 4 = very often). The standard time of response serves as another important factor in the given study. It cannot be quantified in tangible items; however, it is possible to exemplify it through questions. Most importantly, the earlier version of the instrument used in this study has a consistent internal value of 0.90 [4]. Therefore, its coefficient value is 0.88, in line with the current sample.

Ryff Psychological Well-Being Scale (RPWBS): This 54-item scale was used in this study. The RPWBS consists of a series of items reflecting six aspects of psychological well-being (PWB): autonomy, environmental mastery, personal growth, positive relations with others, purpose in life, and self-acceptance [10,11]. The respondents in the study rated items using a six-point Likert scale, ranging from strong disagreement to strong agreement. Sheldon and Lyubomirsky [58] suggested to calculate a total psychological well-being score not by summing the scores for the six subscales but by averaging. In this study, the alpha coefficient for the total score was 0.73. The Lithuanian version of the RPWBS has a reported internal consistency of 0.84 [47].

### 2.4. Procedure

Study participants involved in the survey completed the following instruments: the SSRI, the MSPSS, the PStS-10, and the RPWBS within the planned timelines. The survey exercise was completed within 12 weeks. When conducting the study, the researcher ensured that ethical guidelines were followed, which included legal requirements for conducting the study in Lithuania. Moreover, before involving study participants in the study, permission was sought from the Committee for Social Sciences Research Ethics of the University (protocol No. SMTEK-19, 2018 10 11), who approved the research process. Before taking part in the research study, participants were asked to sign an informed consent.

### 2.5. Data Analysis

**Figure 2:**
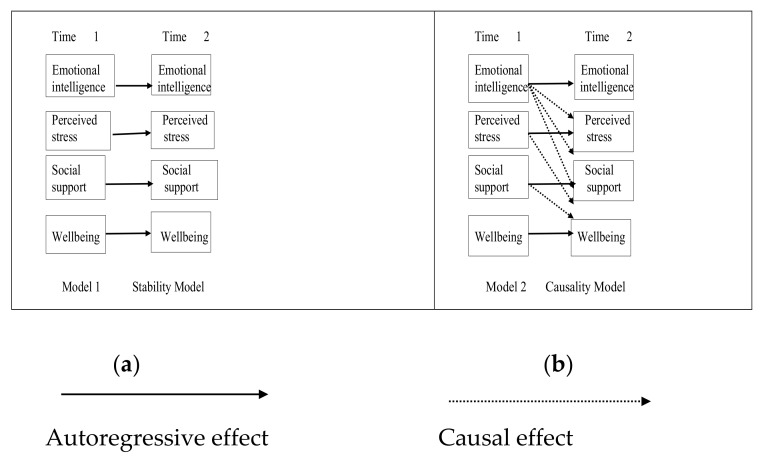
The path model used to assess and examine the path models: (**a**) represents the stability model; (**b**) is an outline of the causality model.

Data collected from this study were analyzed using both descriptive and inferential statistics. The study objectives centered on the assessment of EI associated with other variables, such as perceived social support and stress, undertaken using structural equation modeling (SEM). Mplus version 7.0 (Muthén & Muthén, Los Angeles, CA, USA) was used in this study to ensure that estimation of the variables was provided [59]. Conducting SEM was also integral in testing the significance of the causal processes as demonstrated in Figure 2.

Additionally, the series of structural equation models performed was geared toward finding the best data to match the expected path model. The structural models were then tested using the maximum likelihood estimation method. Therefore, the significance of assessing the two competing full-panel path models helped to ascertain the path model effectively, examine the cross-lagged relations, and prompt a deeper understanding of the causal process, as showcased in the figure below.

Figure 2 showcases the variables as expounded in the objectives of the study. The model adopted for this study provides the baseline levels of the variables in the two timeframes denoted as Time 1 and Time 2. Additionally, the use of autoregressive effects in both cases is key in examining the stability model of the study variables independently and predicting the change in the variables over time. As demonstrated, in the two models the causal relationships manifested between the variables. The indices adopted in the study were conducted using chi-squared, the comparative fit index (CFI), the Tucker–Lewis Index (TLI), the root mean square error of approximation (RMSEA), and the standardized root mean square residual (SRMR), which helped to assess the model’s goodness of fit. As such, the resulting CFI value was 0.95, while the value for TLI was 0.90, with RMSEA recording a value of 0.08. While the SRMR values recorded were lower than 0.05, the χ2/df also recorded a value lower than 3 stipulated by Kline [60], who elaborates on the conditions for an acceptable model fit [61,62].

As one of the main procedures involved in the study, the mediational model involved the relationships between the independent variable (EI), the mediating variables (perceived stress, perceived social support), and the outcome (well-being). The cross-lagged panel model (CLPM) was utilized in the estimation of the two stipulated timelines of the direct and indirect effects. The CLPM consists of two major parts [63], the autoregressive part and the cross-lagged. The autoregressive part measured X, M, and Y at the time (t) using the same variable that was measured the previous time (t – 1). The cross-lagged part was undertaken using longitudinal tests. This was showcased with Path a in the regression of M2, which was measured at Time 2 against X1 controlled at T1. Another estimation was performed using the regression of Y2 against M1 and controlled at Time 1. Path b between M1 and Y2 should be equal to Path b between M2 and Y3. Using this assumption of stationarity, the product ab results in an estimation of the mediational effect of X on Y via M [63].

Despite the findings emanating from the assumptions, the procedure has two shortcomings. Even though we are well positioned to estimate, it is not possible to test the significance of Path c. Similarly, we can test and ascertain whether M is actually a partial mediator, but it is impossible to test if M mediates the X-Y relationship completely [63]. Nevertheless, the mediating factors from perceived social support and perceived stress were tested using the bootstrap estimation procedure. In the current study, a 1000 bootstrap 95% confidence interval (CI) was used to test the significance of the indirect effects. The recommendation of bootstrapping as an analytic approach was approved due to the fact that it involves repeated random sampling observations with the replacement selected from the dataset [64]. The indirect effect measured was found to be significant at the 0.05 level if 95% CI from the bootstrap samples had a missing zero [65].

## 3. Results

### 3.1. Descriptive and Correlation Analyses

Table 1 below showcases means, standard deviations, and Cronbach reliability estimates alongside Pearson correlation coefficients for all variables. Variables defined in this study were checked for skewness and kurtosis, where the values were acceptable as they ranged between −2 and +2, which proves normal distribution and makes it possible to use SEM. Given that there was no correlation that exceeded 0.85, the assumption of multicollinearity was not violated [66]. As predicted, the study participants’ EI correlated positively with perceived social support and well-being at both Time 1 and Time 2. Negative relations were evident between perceived stress and well-being at both Time 1 and Time 2. The two mediating variables (perceived stress and perceived social support) were negatively correlated across the times. As an example, negative relations were evident between perceived stress (at Time 1) and perceived social support (at Time 2; r = –0.25; *p* < 0.01).

The effect size in the study was determined through use of Cohen’s coefficient. Results from this study reported a high correlation between the variables [67].

### 3.2. Mediation Analysis

**Figure 3:**
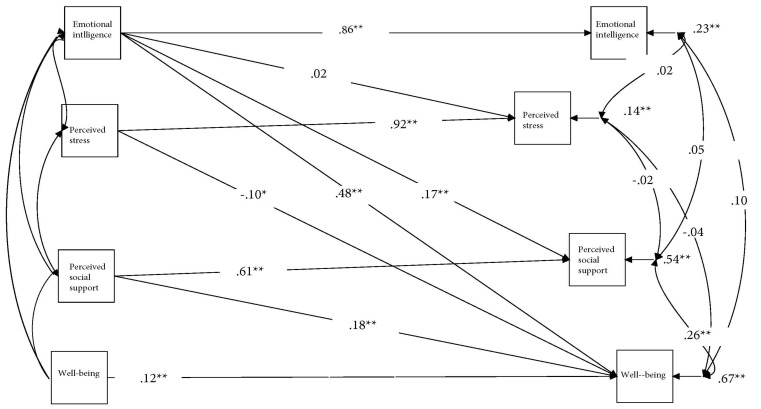
Pathways to mediators and dependent variables. Notes: Values shown are standardized parameter estimates. * *p* < 0.05; ** *p* < 0.01.

In terms of the mediation analysis, the testing of Model 1 was first undertaken alongside the mediating variable. The analysis revealed a poor fit to the data, as demonstrated in the following values: χ2(15, N = 437) = 189.107, *p* < 0.001; CFI = 0.912, TLI = 0.871, RMSEA = 0.163, SRMR = 0.182. Conversely, the causality model (Model 2) reported an acceptable fit to the data: χ2(10, N = 437) = 26.008, *p* < 0.005; CFI = 0.986, TLI = 0.970, RMSEA = 0.061, SRMR = 0.043. The statistics in Figure 3 demonstrate the standardized β weights for the cross-lagged effects in cross-panel mediation analysis. The causal paths were at the 0.01 level; however, this was with the exception of the path formed from EI at Time 1 to perceived stress at Time 2, which was insignificant.

The use of bootstrapping in Table 2 and Figure 3 demonstrates that perceived social support partially mediated the time-specific relationships resulting from the associations (ab = 0.019, 95% CI 0.011–0.040), which were computed at Time 1 and Time 2. Results from the computation of the small size effect [68] confirm the findings reported by Preacher and Kelley [69]. Bootstrapping also reported the association of EI and the associated and mediated variables between Time 1 and Time 2, which was not statistically significant. Additionally, perceived stress only partially mediated the time-specific association between EI at Time 1 and well-being at Time 2. Based on these findings, Hypothesis 1 is not supported. While the time-specific direct effect of EI at Time 1 on well-being at Time 2 was statistically significant, the path of EI on well-being reflected the prediction of change in well-being. However, this was not only controlling for the concurrent correlation at Time 1 but also for the stability of well-being [70].

## 4. Discussion

This study aimed to examine the relationship between EI and psychological well-being in a three-month follow-up study of male university students, taking into account perceived social support and perceived stress as mediators. The findings of this study establish that perceived social support acts as a partially mediating variable in the longitudinal relationship between EI and well-being, but perceived stress does not mediate the longitudinal association between EI and well-being.

The present study has theoretical implications in that a theoretical-hypothetical model for examination was partially confirmed. Our results support only one hypothesis (of two) based on the proposed theoretical-hypothetical model.

Hypothesis 1: We expected that perceived stress would be a mediator in the longitudinal association between EI and well-being. It has been proposed that perceived stress helps researchers to understand emotional intelligence. Therefore, it is important to understand perceived stress pathways into the human mindset in an attempt to reduce risks associated with its reoccurrence. Failure to do so can result in occurrences of anxiety, which affect well-being, as stipulated in Hypothesis 1. The hypothesis was that those male university students with a higher EI quotient have an advantage in experiencing greater support at university, mostly from friends and family members. Notably, greater support has resulted in lower stress levels that necessitate the stabilization of emotional intelligence [5,6]. This has shown positive results associated with the reduction of perceived stress levels [71].

Despite the positive outcomes, individuals that exhibit low levels of emotional intelligence are not able to cope or perform well in a highly stressful environment, which results in anxiety. The hypothesis dictates that perceived stress has a direct role in influencing emotional intelligence in relation to an individual’s well-being [72]. This assertion resulted in the formation of the expectations on the basis of previous studies that were cross-sectional in nature and was concerned with associating the two core variables central to the hypothesis in this study. While the evidence is convincing, our results were not able to support Hypothesis 1.

Based on the results obtained in the previous study [72], there is compatibility associated with university students and workers. It is important to note that two aspects played an integral role in affecting the well-being of participants; burnout and chronic stress affected both the EI and the well-being of an individual. Technically, lack of data about the socioeconomic status of the male students serves as the main setback that limits the ability to generalize. Equally importantly, broadness of the perceived stress construct (PSS is a global measure of perceived stress) did not support the assumptions related to the formulation of the hypothesis in question. Overall, different aspects of perceived stress have reduced while others have increased, resulting in an alteration in terms of longitudinal effect.

Hypothesis 2: This hypothesis argued that perceived social stress is an ideal mediator that confirms the association between well-being and emotional intelligence. The study found that indirect effects and time specificity affected outcomes. While this is the case, the findings mirror what other researchers have studied regarding the area of interest [73,74]. For instance, individuals with higher EI can recognize and manage emotions in others (it helps them manage social situations) and have better possibilities to enhance their social support, which also contributes to an increase in their well-being.

The theoretical contribution to knowledge is that all the results reinforce the findings of previous studies that supported social support functioning as a mediator between EI and well-being indicators associated with happiness and satisfaction in life [2,7,75]. Equally importantly, our findings support all existing social perspectives on EI [35,76,77,78]. The cross-sectional studies that were utilized in the cross-sectional design regarding the two variables do not allow researchers to reach a causal conclusion [23]. The lack of a sufficient timeframe in the research necessitates further research to help establish a link between well-being and emotional intelligence. The sample size for the study serves as a source of strength because it allows consistent sampling to take place, ensuring consistency and validity.

This study also provides important cues for social science theorists and researchers to understand the importance of accounting for perceived social support in longitudinal research concerning perceived stress, EI, and well-being. This study focused on perceived social support being the more powerful factor as compared to perceived stress, which helps to enhance well-being.

Our findings that perceived social support partially mediated the longitudinal association between EI and well-being have practical implications for intervention work designed to build emotional knowledge for males. Those findings have practical implications for training in emotional regulation (training for developing EI) for males. Hence, there is a need to implement EI programs focused on men in educational centers because individuals with higher EI can recognize and manage emotions in others and have better possibilities to enhance their social support, which also contributes to an increase in their well-being.

Limitations associated with this study: In terms of limitations, this study has a number of limitations that affect the accuracy and credibility of the results. Firstly, the study was limited to male university students; thus, there was no consistency, considering that a university sample population encompasses both male and female students. Arguably, the study did not consider the responses of female students, hence creating a bias as far as emotional intelligence is concerned. Nonetheless, the extended sample needs to be employed as further investigation takes place. In the future, scholars need to consider a sample population that captures persons from different genders in a given context and setting to see whether EI is of different importance for the sampled population. This would make it possible to compare the differences in terms of the coefficients between the groups. However, this is not possible here because of the dataset of the current study.

The single self-report measure is the second limitation of the study. In a social context, this implies that social desirability creates a bias in the society that determines the responses of a target group. The CLPM is the tool used in the study, and its underlying assumptions may not hold. For instance, the stationarity assumption in CLPM model may not hold. If an underlying assumption is false, the estimate in question is not practical. Hence, the data will be corrupted, and the hypothesis will not be valid. Furthermore, the data were analyzed without factoring the subscales. This was necessary to obtain outcomes that had total indicators of variables.

Analyzing data without subscales was a limitation because the given SSRI measures were both interpersonal and intrapersonal in nature. It would be interesting from a theoretical perspective to see whether the two facets (intrapersonal and interpersonal aspects) have a differential association with social support, well-being, and stress. The shortcoming demonstrates failure on the part of the researchers to control the variables used in the study. It points to the failure to use an alternative to CLPM. This random intercept cross-lagged panel model (RI-CLPM) was proposed by Hamaker, Kuipers, and Grasman [79]. The RI-CLPM stipulates that each observed score was split between a person part and a within-person part, which requires the use of more than two waves of data. Hence, the CLPM was ideal in the study because it required two sets of wave data. 

Scholars conducting similar research need to consider different tactics that mediate the effects of perceived stress and the impacts of social support. The findings should be contrasted with how these variables affect emotional intelligence. With the lack of three waves, it is not possible to test for serial mediation showing the correlation between social support and perceived stress among male students within the university. This needs to be done in in longitudinal studies with three or more time lags that exist between psychological well-being and emotional intelligence.

Future directions: It is imperative to have more longitudinal studies that have more and diverse time lags and different populations. Notably, further research needs to be conducted to investigate other mediators that are likely to affect EI and well-being in terms of the longitudinal association between the two variables. Other variables might also be vital in mediating the possible effects of EI on well-being in terms of pessimism and optimism, which are important and useful predictors in determining the psychological well-being of individuals [3,80]. Despite the continued research, which is vital to support the evidence base that is important in understanding emotional intelligence, this variable could be the focus of future research aimed at enhancing psychological well-being among males studying in university. All these measures are in place to help foster better performance by teaching the student the survival tactics necessary to thrive in a challenging environment.

## 5. Conclusions

The findings in this study emerged through the use of structural equation modelling, which evidenced the relationship between EI and well-being. It was established that perceived social support partially mediated the longitudinal association between EI and well-being. Specifically, perceived stress did not mediate the association between EI and well-being over time. The results in this study are supported by evidence reported in previous studies, as demonstrated in the discussion. In addition to the relationship reported between emotional intelligence and well-being, findings from this study highlight the significance of sustaining the psychological well-being of individuals.

## Figures and Tables

**Table 1:** Means, standard deviations, and correlations between the study variables.

Variable	*M*	SD	1	2	3	4	5	6	7	8
1. EI (T1)	3.77	0.29	(0.86)							
2. EI (T2)	3.78	0.30	0.78 **	(0.86)						
3. PS (T1)	2.31	0.67	–0.19 **	–0.19 **	(0.89)					
4. PS (T2)	2.32	0.69	–0.16 **	–0.16 **	0.73 **	(0.88)				
5. PSS (T1)	4.53	0.37	0.16 **	0.21 **	–0.15 **	–0.15 **	(0.70)			
6. PSS (T2)	4.48	0.37	0.29 **	0.31 **	–0.25 **	–0.24 **	0.65 **	(0.71)		
7. WB (T1)	5.26	0.22	0.12 **	0.14 **	–0.08	–0.09	0.11 *	0.10 *	(0.72)	
8. WB (T2)	5.16	0.21	0.53 **	0.52 **	–0.24 **	–0.23 **	0.28 **	0.43 **	0.14 **	(0.72)

Notes: Scale reliabilities are indicated in parentheses on the diagonal. EI = emotional intelligence score; PS = perceived stress score; PSS = perceived social support score; WB = well-being score; T1 = Time 1; T2 = Time 2. * *p* < 0.05, ** *p* < 0.01.

**Table 2:** Bootstrap estimates of the cross-lagged (time-specific direct) and mediational (time-specific indirect) effects.

Model Pathways	Parameter Estimate	Bias-Corrected CI (95%)	
	Unstandardized (*SE*)	Lower	Upper
Cross-lagged effect of EI (T1) on well-being (T2)	0.340 (0.037)	0.268	0.410 *
Indirect effect via PS (T1) of EI (T1) on well-being (T2)	−0.001 (0.001)	−0.007	0.001
Indirect effect via PSS (T1) of EI (T1) on well-being (T2)	0.019 (0.007)	0.011	0.040 *

Notes: *N* = 437. EI = emotional intelligence; PSS = perceived social support; PS = perceived stress; T1 = Time 1; T2 = Time 2; CI = confidence interval; SE = standard error. * This 95% confidence interval excludes zero; therefore, the relationship is significant at *p* < 0.05.
